# Depressive Symptoms Are Associated With Increased Healthcare Utilization Among Older Adults in Singapore

**DOI:** 10.1093/geroni/igaf122.214

**Published:** 2025-12-31

**Authors:** Rahul Malhotra, Abhijit Visaria, Rakhi Vashishtha, Qiqi Cheng, Angelique Chan

**Affiliations:** Duke-NUS Medical School, Singapore, Singapore; Duke-NUS Medical School, Singapore, Singapore; Centre for Behavioural and Implementation Science Interventions (BISI), Yong Loo Lin School of Medicine, National University of Singapore, Singapore, Singapore; University of Manchester Manchester Institute of Education, Manchester, England, United Kingdom; University of Manchester Manchester Institute of Education, Manchester, England, United Kingdom

## Abstract

Population ageing presents a significant challenge to healthcare systems, as older adults typically have higher rates of healthcare utilization. This study investigated the impact of depressive symptoms on healthcare utilization among community-dwelling older adults in Singapore, focusing on emergency department (ED) visits, frequent ED use (≥4 visits), and inpatient hospitalizations. We analyzed data from a nationally representative longitudinal cohort with participants’ survey data linked to electronic medical records to objectively measure healthcare utilization outcomes. We utilized two-part models for ED visits and hospitalizations and logistic regression for frequent ED use. We observed that higher depressive symptoms were associated with increased odds of ED visits and hospitalizations within 12 months. Each unit increase in the depressive symptoms score was linked with 5% higher odds of at least one ED visit and 6% higher odds of hospitalization. Furthermore, depressive symptoms were associated with a 13% increase in the odds of frequent ED use. This study provides robust evidence on the longitudinal association of depressive symptoms with healthcare utilization among older adults, specifically in an Asian population. Given the growing older adult population and the significant healthcare costs associated with hospitalizations and ED visits, these findings highlight the need for proactive identification and management of depressive symptoms in older adults to potentially reduce future healthcare burdens.

